# EbolaID: An Online Database of Informative Genomic Regions for Ebola Identification and Treatment

**DOI:** 10.1371/journal.pntd.0004757

**Published:** 2016-07-21

**Authors:** João Carneiro, Filipe Pereira

**Affiliations:** Interdisciplinary Centre of Marine and Environmental Research (CIIMAR), University of Porto, Porto, Portugal; University of Minnesota, UNITED STATES

## Challenge

The Ebola virus disease (EVD) is a rare and deadly disease affecting humans and other primates caused by infection with a virus of the family *Filoviridae*, genus *Ebolavirus* [1]. The March 2014 Ebola epidemic is the largest in history, affecting multiple countries in West Africa, with more than 11,301 deaths reported by the World Health Organization by March 2016. Methods based on polymerase chain reaction (PCR) are commonly used for virus detection with the advantage of requiring low amounts of viral samples, minimal manipulation, and minimal equipment. In particular, quantitative PCR (qPCR) has been used for the sensitive and fast identification of patients with EVD [2–7]. However, the high genetic diversity of RNA viruses poses a challenge for the design of efficient nucleic acid-based assays, as suggested by the high substitution rate observed in Ebola virus from the 2014 outbreak [5,8]. Recent studies suggest that false-negative diagnosis or inefficient therapeutics can occur due to sequence variation at binding sites of PCR primers, probes, small interfering RNAs (siRNAs), or monoclonal antibodies targeting the Ebola virus [5,9]. For this reason, the selection of reliable oligonucleotides and target genomic regions for use in nucleic acid-based assays is crucial for the correct diagnosis and treatment of EVD.

## The EbolaID Database

**Fig 1:**
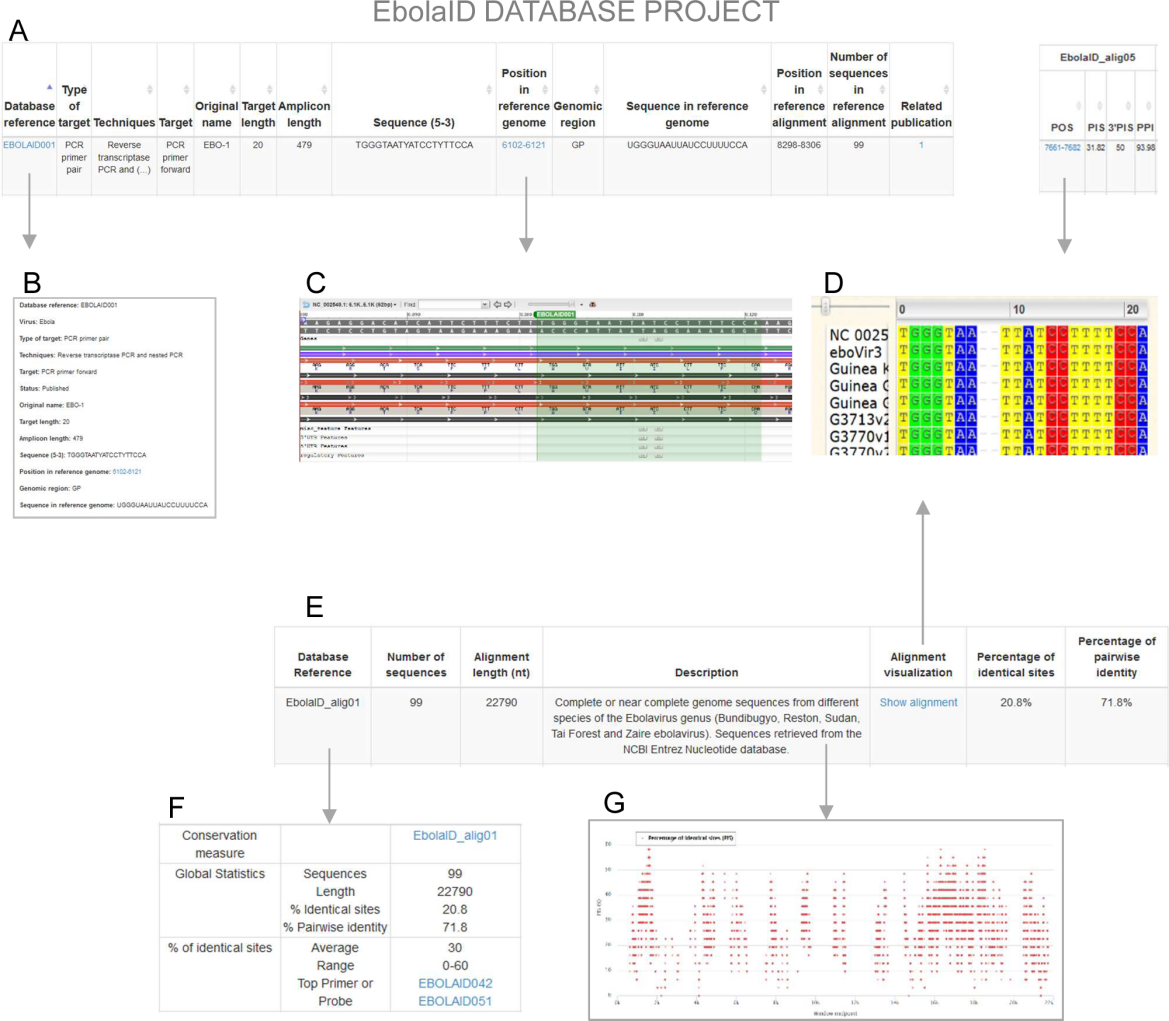
Screenshot of the data and tools included in the EbolaID database. A) Section with the list of oligonucleotides. B) Section with detailed information for each oligonucleotide. C) NCBI sequence viewer of reference genome with oligonucleotide annotation and feature tracks. D) Wasabi alignment viewer of a genomic region. E) Table with description of a multiple sequence alignment. F) Summary of different conservation measures for an alignment. G) Scatter plot describing a sliding window analysis of diversity measures across the Ebola genome.

The EbolaID database (http://ebolaid.portugene.com) is a free, web-accessible database built to facilitate the design of accurate molecular methods for detection and identification of the Ebola virus (Fig 1). The database provides an interface for browsing, filtering, and downloading data from published oligonucleotide sequences (PCR primers, real-time PCR probes, etc.) annotated according to a reference genome. The user can find varied information for each oligonucleotide: the type of technique where it was originally used, the sequence, location in the reference genome, references, etc. The database provides different measures of sequence conservation for each target region obtained from multiple sequence alignments, allowing the selection of the most conserved oligonucleotides. The sequences can be visualized, edited, and exported using the Wasabi (http://wasabi2.biocenter.helsinki.fi/) and NCBI (https://www.ncbi.nlm.nih.gov/tools/sviewer/) sequence viewers. The database also describes the most conserved and variable regions of the Ebola genome for those researchers interested in designing new oligonucleotides. Box 1 discusses the advantages and disadvantages of the database.

**Box 1. Advantages and Disadvantages of the EbolaID Database**

**Advantages**

- The use of different multiple sequence alignments and measures of sequence conservation
- Detailed information on each oligonucleotide with links to references
- Query tools for selecting and evaluating the oligonucleotides

**Disadvantages**

- Restricted to oligonucleotides described in peer-reviewed publications
- Large tables with numeric values can be difficult to visualize
- Data from different publications may have different levels of reliability

## Database Coverage

The EbolaID database was constructed using information from 17 peer-reviewed works published from 1999 to 2014. Currently, the database includes 57 oligonucleotides (48 primers and 9 probes) retrieved from publications describing assays for virus detection or from epidemiological and phylogenetic studies [8,10–12]. The oligonucleotides included in the database are located in the NP (33.3%), GP (21.0%), L (38.6%), VP24 (3.5%), and 3′UTR (3.5%) regions of the Ebola genome. The database includes multiple sequence alignments of complete or nearly complete genomic sequences of ebolaviruses and marburgviruses used to estimate the level of sequence conservation in oligonucleotides (a description of the alignments can be found in the “Sequence alignments” section of the website). For example, the database includes an alignment with 99 sequences from different species of the *Ebolavirus* genus (Bundibugyo, Reston, Sudan, Tai Forest, and Zaire ebolavirus) retrieved from the NCBI Entrez Nucleotide database (http://www.ncbi.nlm.nih.gov). The recent 2014 outbreak is represented by an alignment with 81 sequences from 2014 plus 20 sequences from earlier outbreaks (from 1976 to 2008) built by Gire et al. [8].

### Selecting the Best Oligonucleotides

The EbolaID database provides different measures of sequence conservation for each oligonucleotide (details at http://ebolaid.portugene.com/EbolaID_definitions.html): a) percentage of identical sites (PIS), calculated by dividing the number of equal positions in the alignment for an oligonucleotide by its length; b) percentage of identical sites in the last five nucleotides at the 3′ end of oligonucleotides (3′PIS); and c) percentage of pairwise identity (PPI), calculated by counting the average number of pairwise matches across the positions of the alignment. The oligonucleotides can be selected by considering a ranking score (“EbolaID score”) with the mean value of the three different measures (PIS, 3′PIS, and PPI). In general, the oligonucleotides with the highest values for these measures have a higher probability of binding to the target genomic region, increasing the probability of a positive detection. The three oligonucleotides with the highest EbolaID score (above 88) are located in the L gene (EBOLAID027, EBOLAID051, and EBOLAID036) and are part of the most conserved PCR primer pairs. All data can be accessed through the “Search” section of the database.

## Example of Use

Here we describe a hypothetical situation where a researcher needs an oligonucleotide for the detection of the Ebola viruses of the recent 2014 outbreak. The user can start by accessing the “Sequence alignments” tab at the top of the EbolaID homepage, which opens a table describing all sequence alignments included in the database. The user can visualize each alignment and progress to the table with the list of all oligonucleotides ordered according to the degree of conservation. Alternatively, the researcher can go to the “Search” tab and select “Oligonucleotides in specific alignments”, which describes different measures of sequence conservation for each oligonucleotide and alignment. The list of oligonucleotides can be ordered by the PIS or PPI measures. For example, the user will find that the most conserved oligonucleotide currently in our database for the “EbolaID_Alig03” alignment is EBOLAID039. The most conserved oligonucleotides can also be found in the “The top alignment” section of the search page. All details on the selected oligonucleotide, including the sequence, genomic location, and references, can be found by clicking on the primer name (Fig 1). The researcher can also access the list with the most conserved genomic regions in each alignment in the top menu named “Genome variation” or in the table at the “Sequence alignments” section.

## Conclusion

Although several online resources include information on the *Ebolavirus* genus, the EbolaID database is by far the largest set of oligonucleotides currently available for this group of viruses. For example, the Hemorrhagic Fever Viruses (HFV) database [13] has curated nucleotide and protein sequence alignments describing the species and outbreak associated with each Ebola virus record but lacks information on target regions for molecular methods. The Virus Pathogen Database and Analysis Resource (ViPR) and the NCBI *Ebolavirus* databases [14,15] are very useful resources with multiple sequence alignments and phylogenetic trees but were not designed to guide the design of molecular methods. The EbolaID database can help researchers interested in designing accurate assays for the identification of the Ebola virus or to select conserved genomic regions for sequence-based therapeutics. The annotated reference genome and multiple sequence alignments can also be useful for epidemiological and phylogenetic studies.
